# Application of the multicellular tumour spheroid model to screen PET tracers for analysis of early response of chemotherapy in breast cancer

**DOI:** 10.1186/bcr1747

**Published:** 2007-07-22

**Authors:** Azita Monazzam, Raymond Josephsson, Carl Blomqvist, Jörgen Carlsson, Bengt Långström, Mats Bergström

**Affiliations:** 1 Institute of Oncology, Institute of Oncology, Radiology and Clinical Immunology, Uppsala University Hospital, SE-751 85 Uppsala, Sweden; 2 Uppsala Imanet, GE Healthcare (PET Center), SE-751 09, Uppsala, Sweden; 3 Clinical Imaging, Novartis Pharma, CH-4002, Basel, Switzerland; 4 Department of Biomedical Radiation Sciences, Institute of Oncology, Radiology and Clinical Immunology, Uppsala University Hospital, SE-751 85 Uppsala, Sweden; 5 Department of Pharmaceutical Biosciences, Uppsala University, SE-751 24, Uppsala, Sweden

## Abstract

**Introduction:**

Positron emission tomography (PET) is suggested for early monitoring of treatment response, assuming that effective anticancer treatment induces metabolic changes that precede morphology alterations and changes in growth. The aim of this study was to introduce multicellular tumour spheroids (MTS) to study the effect of anticancer drugs and suggest an appropriate PET tracer for further studies.

**Methods:**

MTS of the breast cancer cell line MCF7 were exposed to doxorubicin, paclitaxel, docetaxel, tamoxifen or imatinib for 7 days for growth pattern studies and for 3 or 5 days for PET tracer studies. The effect on growth was computed using the semi-automated size determination method (SASDM). The effect on the uptake of PET tracers [^18^F]3'-deoxy-3'-fluorothymidine (FLT), [1-^11^C]acetate (ACE), [^11^C]choline (CHO), [^11^C]methionine (MET), and 2-[^18^F]fluoro-2-deoxyglucose (FDG) was calculated in form of uptake/viable volume of the MTS at the end of the drug exposures, and finally the uptake was related to effects on growth rate.

**Results:**

The drugs paclitaxel, docetaxel and doxorubicin gave severe growth inhibition, which correlated well with inhibition of the FLT uptake. FLT had, compared with ACE, CHO, MET and FDG, higher sensitivity in monitoring the therapy effects.

**Conclusion:**

SASDM provides an effective, user-friendly, time-saving and accurate method to record the growth pattern of the MTS, and also to calculate the effect of the drug on PET tracer uptake. This study demonstrate the use of MTS and SASDM in combination with PET tracers as a promising approach to probe and select PET tracer for treatment monitoring of anticancer drugs and that can hopefully be applied for optimisation in breast cancer treatment.

## Introduction

Positron emission tomography (PET) has demonstrated usefulness in monitoring therapeutic response in a wide range of cancers, including breast cancer [1,2]. This is because effective therapy leads to rapid physiological modification in tumours; with the right choice of PET tracer, this modification can easily be revealed and the therapeutic response can be clarified [3]. Physicians can thus quickly modify less effective therapy, thereby improving patient outcomes and reducing the cost of ineffective treatment.

To date, 2-[^18^F]fluoro-2-deoxyglucose (FDG) has been the most common PET tracer; however future applications of PET will likely involve other tracers to improve characterisation of tumour biology and more effectively measure response to therapy [4,5]. This potential refinement in tumour characterisation will help to predict clinical behaviour and tailor therapy of tumour biology, thereby individualising treatment.

In preclinical investigations, the multicellular tumour spheroid (MTS) system has provided an appropriate *in vitro *system to evaluate and predict tumour response to chemotherapy agents [6-8]. It has been clearly shown that MTS are functionally and physiologically superior to monolayer cultures [9-14]. Another important advantage of MTS is the possibility for long-term follow up. We have previously shown that MTS in combination with the semi-automated size determination method (SASDM) [15] is suitable for preclinical studies of PET tracer uptake, with simplified handling during incubation, washing and measurements [16].

This study aimed to illustrate the practicality of the method and to indicate the potential to correlate drug effects on PET tracer uptake (short-term effect) with their effects on spheroid growth (long-term effect) by including five different anticancer drugs and five different PET tracers. This introduces the possibility of using the MTS model in combination with SASDM as an applicable preclinical method to screen a range of PET tracers for breast cancer treatment monitoring.

## Materials and methods

### Cell culture

Cells of the MCF-7, oestrogen receptor positive human breast cancer line (European Collection of Cell Cultures, Salisbury, UK) were grown in MEM/EBSS supplemented with 10% FCS, 1 mM sodium pyruvate, 2 mM L-glutamine, 1% non-essential amino acids and 5% penicillin (Tamro, Vantaa, Finland). The medium was changed twice weekly and cells were maintained in exponential growth phase.

### Multicellular tumour spheroids

The tumour cells were trypsinised from the stem monolayer culture. Cell suspensions were then seeded in 24-well, 1% agarose-coated culture plates, with approximately 50000 cells per well. MTS were grown in DMEM (high glucose) supplemented with 10% FCS, 1 mM sodium pyruvate, 2 mM L-glutamine, 1% non-essential amino acid, 5% penicillin, 0.01 mg/ml insulin and 1 nM β-estradiol (Tamro), and were kept at 37°C with 5% CO_2_. After 6 days, stable spheroids had formed that were approximately 1.2 mm in diameter.

### Anticancer drug treatment

The five anticancer agents: paclitaxel, docetaxel, doxorubicin, tamoxifen and imatinib were obtained from Novartis (Basel, Switzerland) and diluted in the growing culture medium to a concentration of 1 μM or 10 μM. The recommended dose for patients is in the range of 1–10 μM, if an equal distribution of the drug in body is assumed. Treatment was initiated at day 6 of MTS growth with changing the culture medium to the drug-containing medium. After 1 h the medium with drug was renewed to ensure adequate mixing.

In each experimental set-up, four MTS in one 24-well plate were referred to as one group. The groups included control (without treatment agent) and five different treatment drugs with the same concentration.

The response to each anticancer treatment was evaluated by measurement of the tracer uptake and viable volume of the MTS after 3 and 5 days of treatment. Additionally, each spheroid was photographed before and during drug treatment using a Nikon Colorpix 4500 (Nikon, Tokyo, Japan) camera mounted on an inverted Zeiss microscope (Carl Zeiss MicroImaging, Inc. Thornwood, New York, USA). The digital images were stored for evaluation of size.

### Semi-automated size determination method (SASDM)

Digital images of MTS were evaluated with respect to area of total spheroid, rim of viable cells and central necrosis using SASDM software, as previously described [15]. Knowing the area of total spheroid and area of central necrosis, the respective volumes were calculated assuming the third dimension to be described by the geometric mean of the diameters of an ellipsoid with the same area as determined. Finally the volume of viable cells was calculated as the difference between the total volume and the volume of the necrosis.

### PET tracers

To monitor the effect of each anticancer treatment five established PET tracers were used: [1-^11^C]acetate, [^11^C]choline, [^11^C]methionine, [^18^F]3'-deoxy-3'-fluorothymidine and 2-[^18^F]fluoro-2-deoxyglucose. All were synthesised immediately prior to use at the PET centre of Uppsala Imanet according to standard procedures.

### PET tracer uptake

The MTS were incubated for 50 min (for ^18^F-labelled tracers) and 30 min (for ^11^C-labelled tracers) at 37°C with 0.5 ml medium per well containing 3 MBq tracer, and then washed 3 × 5 min (for ^18^F-labelled tracers) and 3 × 3 min (for ^11^C-labelled tracers) with medium (1 ml/well). Finally, MTS with 20 μl washing medium were transferred to 5 ml tubes and tracer uptake was measured in a calibrated well γ-counter. A 20 μl sample of the incubation medium was measured as reference, and 20 μl from the last wash medium was measured as background control.

For each spheroid in each treatment group the total tracer uptake (activity concentration (Bq/ml)) and the tracer uptake per viable volume MTS (activity concentration (Bq/ml)/viable volume (mm^3^)) were determined, and the averages over the treatment groups were normalised to the control group. Each experimental set-up was repeated three times.

### Dose effect study

Based on the PET screening and dose-dependence result, the dose effect of docetaxel and doxorubicin were investigated, using [^18^F]3'-deoxy-3'-fluorothymidine (FLT) as tracer.

The MTS were treated with different doses of docetaxel (1, 3, 5, 10 or 20 μM) and doxorubicin (0.1, 1, 3, 5, or 10 μM) and then the FLT uptake in the MTS were measured after 3 days of treatment.

### Growth pattern study

The effect of each chemotherapy agent on growth pattern was investigated. The size determinations included assessment of total MTS volume, including central necrosis, and viable cell volume in which case only the rim of viable cells were included. The relative volumes were presented in percent of starting volume and presented in relation to days after start of treatment.

To determine effect on growth, the following calculations and illustrations were made. The growth rate for each dose group was calculated as:

τ = 0.693/Td

where the growth pattern was fitted to the equation:

V = V_0_*Exp(τ*t).

This equation assumes exponential growth, starting with the initial volume of V_0_, and with a doubling time of Td.

Effect on growth rate was expressed as:

100*(τ_0_-τ_D_)/τ_0_

where τ_0 _denotes growth rate without drug and τ_D _denotes growth rate with drug. This indicated that lack of drug effect is given as 0%, total growth cessation as 100% and regression as a value larger than 100%.

The reduction in tracer uptake in MTS was defined as:

100*(U_0_-U_D_)/U_0_

where U_0 _represents activity concentration (Bq/ml)/viable volume (mm^3^) of MTS without treatment and U_D _represents activity concentration (Bq/ml)/viable volume (mm^3^) of MTS with treatment.

Finally, the effect of each drug in form of growth retardation and reduction in PET tracer uptake was correlated.

### Ki67 staining

Proliferation of tumour cells within spheroids was assessed by Ki67 staining. At 3 days after treatment spheroids were washed in medium, fixed in 4% paraformaldehyde and placed in paraffin blocks. Serial 4-μm sections of the paraffin blocks were cut using a cryotome and mounted on poly-L-lysine-coated slides that were fixed in hydrogen peroxide and 90% methanol. Ki67 staining was performed using a monoclonal mouse antibody directed against Ki67 and MOM kit (Vector Labs, Burlingame, CA, USA). Images were captured digitally using a Nikon Colorpix 4500 camera mounted on an inverted Zeiss microscope.

## Results

### Drug-induced growth disturbances

All drugs, except imatinib, showed a clear effect in the form of growth rate reduction or even regression of size of MTS. In MTS treated with 10 μM imatinib, a slight growth enhancement was observed. Growth inhibition observed in MTS treated with doxorubicin and tamoxifen showed more apparent dose dependence compared to the taxanes (Table 1).

**Table 1:** The effect of chemotherapy agents on growth pattern

**Treatment**	**Dose (μM)**	**% Growth inhibition**
Paclitaxel	1	141
	10	200
Docetaxel	1	126
	10	144
Doxorubicin	1	188
	10	360
Tamoxifen	1	36
	10	127
Imatinib	1	0
	10	-12

### Uptake of the PET tracers

The relative uptake (activity in viable part of the MTS/activity in incubation medium) of all PET tracers was in the range of 1–1.5. However, this relative moderate uptake in relation to medium does not translate into a moderate contrast *in vivo*, as there the background is washed out significantly with time. The uptake of the PET tracers after 3 days of drug exposure is shown in Table 2.

**Table 2:** The effect of chemotherapy agents on tracer uptake after 3-day treatment

**Treatment**	**Dose (μM)**	**% PET Tracer uptake reduction**				
		
		**FLT**	**FDG**	**CHO**	**MET**	**ACE**
Paclitaxel	1	68 ± 7	21 ± 8	-13 ± 9	6 ± 7	-10 ± 7
	10	69 ± 7	17 ± 7	-6 ± 9	7 ± 7	-10 ± 8
Docetaxel	1	66 ± 6	27 ± 7	-3 ± 7	13 ± 6	1 ± 5
	10	66 ± 6	27 ± 6	-2 ± 7	1 ± 6	-7 ± 5
Doxorubicin	1	76 ± 5	18 ± 5	5 ± 6	-8 ± 5	14 ± 5
	10	95 ± 6	71 ± 6	27 ± 8	38 ± 9	48 ± 7
Tamoxifen	1	11± 8	2 ± 9	-2 ± 9	0 ± 7	-7 ± 7
	10	28 ± 7	22 ± 8	-2 ± 9	-7 ± 7	-45 ± 7
Imatinib	1	7 ± 7	3 ± 9	4 ± 9	-6 ± 7	2 ± 7

#### Paclitaxel

In MTS treated with paclitaxel, an inhibitor of microtubule remodelling, FLT uptake decreased by about 70%. FDG uptake decreased slightly but not as much as FLT, and alteration in [^11^C]choline (CHO), [^11^C]methionine (MET) and [1-^11^C]acetate (ACE) uptake was insignificant.

#### Docetaxel

In MTS treated with docetaxel, also an inhibitor of microtubule remodelling, FLT uptake decreased by about 70%. FDG uptake decreased slightly but not as much as FLT, and alteration in CHO, MET and ACE uptake was insignificant. PET tracer uptake showed a similar pattern as in MTS treated with paclitaxel.

#### Doxorubicin

In MTS treated with doxorubicin, an anthracycline antibiotic, FLT uptake decreased considerably by about 90% but uptake of FDG, CHO, MET and ACE decreased less.

#### Tamoxifen

In MTS treated with tamoxifen, an anti-oestrogen, significant tracer uptake alteration was observed only in the 10 μM dose. FLT and FDG uptake decreased by about 20%, CHO and MET uptake was barely affected, whereas ACE uptake increased by about 40%.

#### Imatinib

In MTS treated with imatinib, a protein-tyrosine kinase inhibitor, tracer uptake alteration was observed only with the 10 μM dose. FLT uptake was hardly affected, FDG and CHO uptake decreased by about 20% and MET and ACE uptake was not significantly changed.

### Correlation between tracer uptake and growth

**Figure 1:**
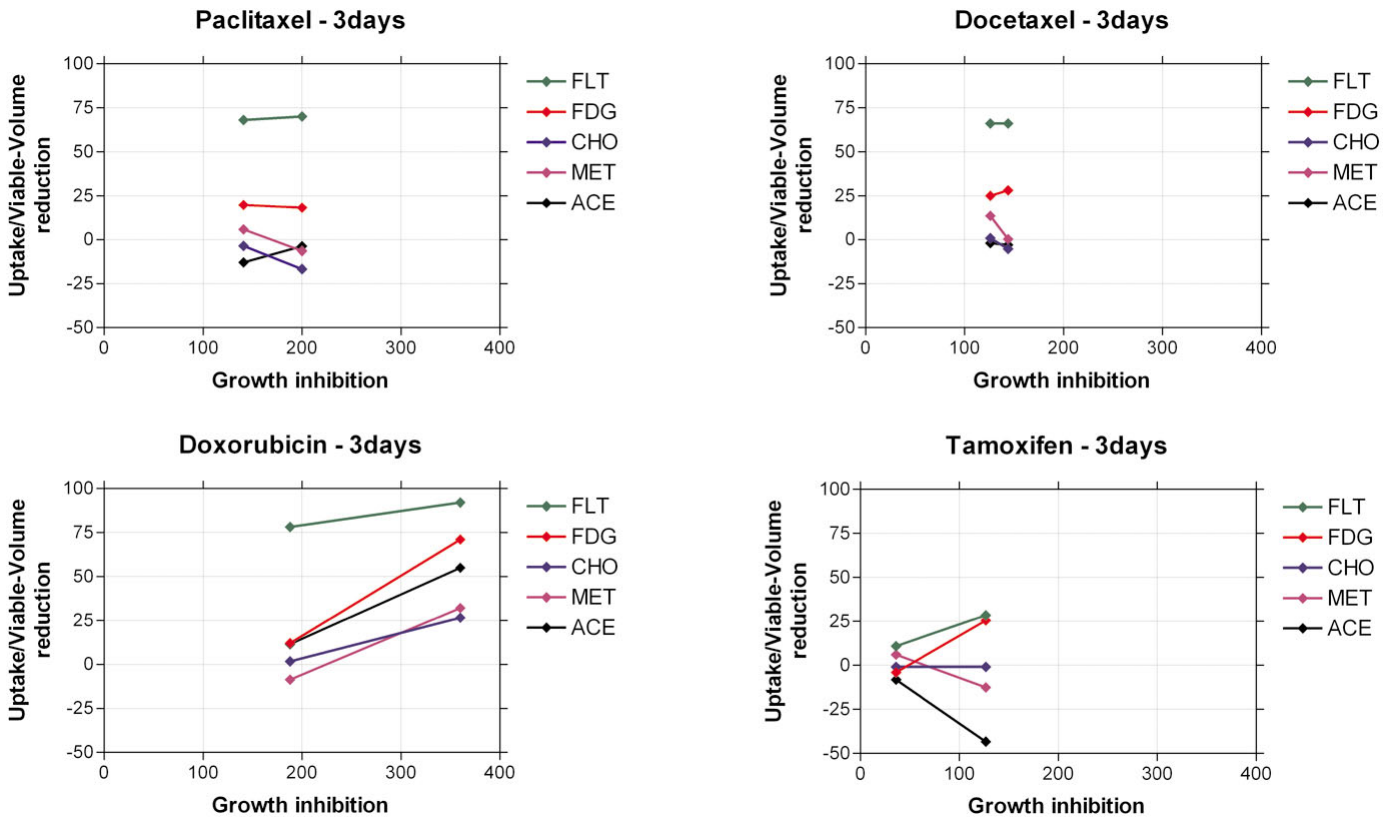
Correlation between growth inhibition and tracer-uptake reduction after 3 days of treatment (1 and 10 μM).

The drug-induced changes in uptake of PET tracers as a function of the drug-induced growth disturbances are plotted in Figure 1. FLT seemed to be the most appropriate PET tracer for treatment follow-up of MTS. FLT responded with reduction in uptake shortly after treatment in correlation with the growth inhibition. A reduced growth rate of more than 100% was associated with more than 70% reduction in FLT uptake for paclitaxel, docetaxel and doxorubicin. However, on treatment with tamoxifen, a growth inhibition was noted in spite of only 20% reduction of FLT uptake.

For FDG, the high reduction of growth rate was associated with about 25% reduction in tracer uptake in spheroids treated with taxanes, doxorubicin and 10 μM tamoxifen. The uptake of the tracers CHO, MET and ACE showed small changes or in some cases even increases in spite of significant growth inhibition effects by the drugs.

### Dose effect of docetaxel and doxorubicin

**Figure 2:**
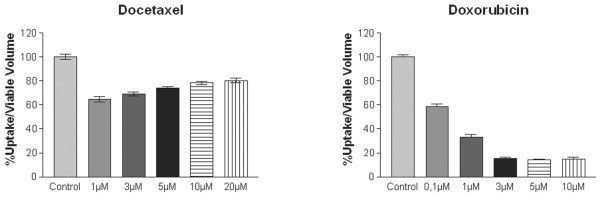
The dose effect of treatment with docetaxel and doxorubicin recorded with FLT.

In a separate study on the dose effects, FLT uptake decreased gradually with increased doses of doxorubicin (Figure 2). This was in contrast to the results after docetaxel exposure.

The experiment suggests that the maximum effect of docetaxel occurs below 1 μM, which is consistent with the potential that docetaxel saturates its target. The slight increase with dose observed could relate to other phenomena exerted by docetaxel, e.g. related to changes in cellular contact.

The results were consistent with the effects on growth where a minor dose relation was observed with docetaxel whereas a major dose relation was observed with doxorubicin (Table 1). However, the effect of docetaxel in FLT uptake in these experiments was more moderate than the effect observed in the experiments performed earlier (PET tracer uptake experiments). The explanation is likely related to the different conditions of the experiments, e.g. different batches of the MCF-7 cell line and different batches of medium.

### Immunohistochemistry

**Figure 3:**
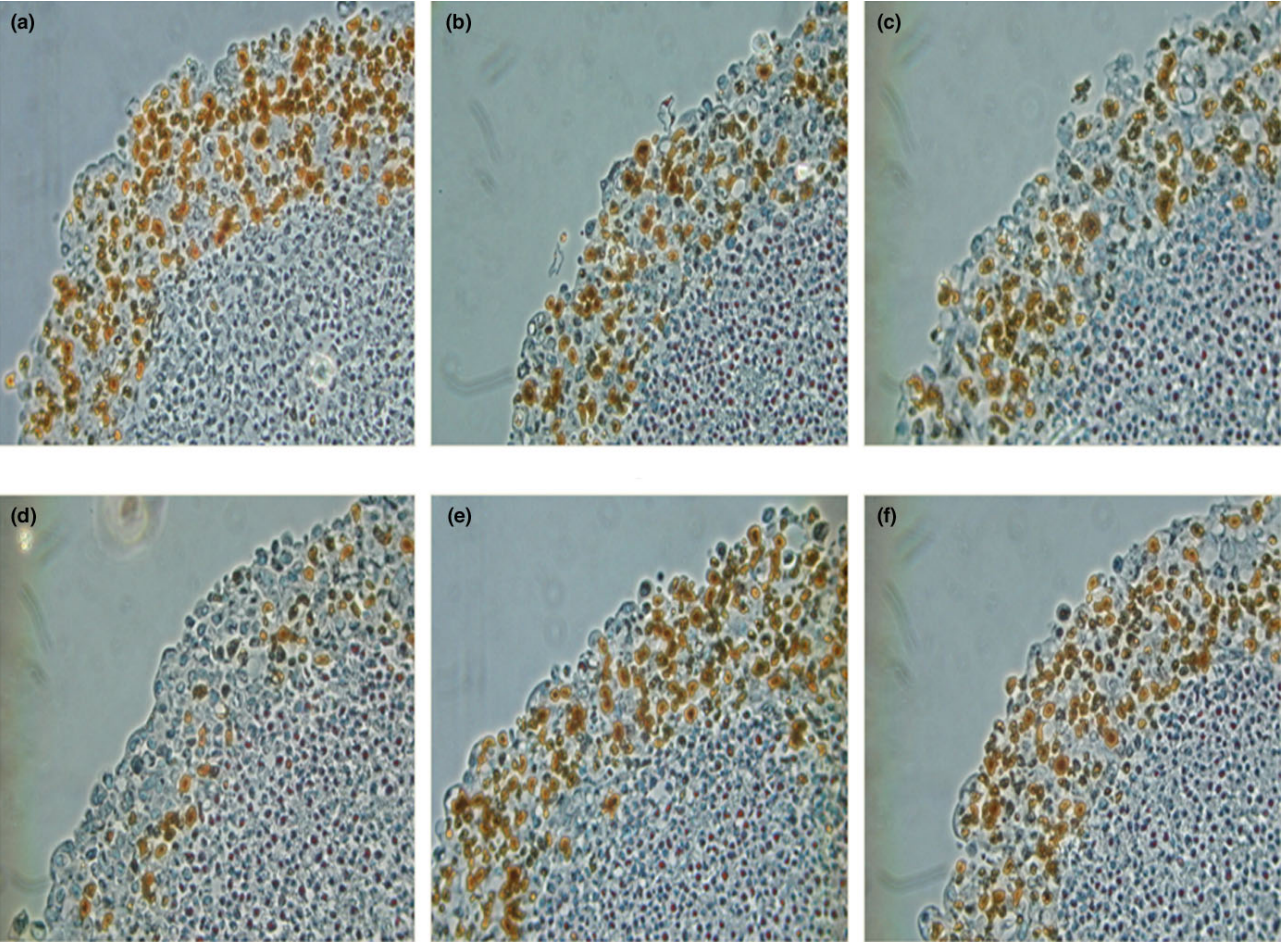
Ki67 staining of spheroid sections illustrating proliferating cells (black colour) after 3-day exposure to 1 μM non-drug medium **(a)**, paclitaxel **(b)**, docetaxel **(c)**, doxorubicin **(d)**, tamoxifen **(e) **and imatinib **(f)**.

Ki67 staining of spheroid sections depicted a decrease in proliferating cells in MTS treated with taxanes and doxorubicin (Figure 3).

## Discussion

Effective chemotherapy is expected to induce various metabolic changes in tumours that are followed by alterations in morphology and growth inhibition, unregulated cell death or apoptosis. It can be assumed that the physiological changes occur before changes in the tumour size can be observed. This is the principle behind the concept that an efficient chemotherapy can be detected with PET at earlier stages than with other types of imaging modalities. However, the question remains which metabolic modifications will occur after a specific chemotherapy, and which PET tracer is most sensitive in detecting those modifications. Of equal importance is ascertaining when a treatment induces metabolic changes that are recordable with PET that do not translate into an anti-tumoural action such as inhibition of growth.

Although there is a relatively good understanding of the mechanisms by which certain anticancer drugs exert their effect, and it is relatively well known by which mechanisms certain PET tracers accumulate in tumour cells, it is usually less obvious how these mechanisms are interrelated. The correlation is especially difficult to predict with PET tracers that reflect rather general physiological phenomena, e.g. there is no obvious connection between microtubule inhibition and expression of glucose transporters. Hence, without an understanding of these links, we must revert to empirical evaluations of correlations. This increases the complexity of the situation, as correlations observed in a certain assay with its own experimental conditions might not be relevant for other conditions. For example, observations made with a certain time of drug exposure might not be reproduced with another drug schedule. In this sense, we believe that the proposed assay is an important methodological contribution because it allows a work-efficient way of exploring different experimental schemes, and the spheroid model allows variable scheduling to be simulated.

The aim of the present study was to explore our assumptions and to complement our previous studies on use of MTS and monitoring of PET tracer uptake by illustrating its ease and potential using five different drugs and five different PET tracers available at multiple PET centres, all of which have shown usefulness in the diagnosis of different types of cancer. Of special importance has been the use of the semi-automated size determination method (SASDM), as it allows an accurate and precise assessment of the volume of viable cells in the spheroids. This in turn gives us an opportunity to define tracer uptake in relation to viable volume and not only the total volume, which is especially important in long-term studies and drug effect studies where the proportion between viable volume and necrosis can be expected to change. Furthermore, it allows a better estimate of growth rate and effect on growth rate by treatment.

The use of ACE in PET has been suggested in urologic malignancies, hepatocellular carcinoma (HCC) and cardiac disease. The mechanism of tumour retention of this tracer differs from that of myocardium, in which ACE is incorporated into the citric acid cycle and rapidly metabolised. The dominant process of ACE incorporation in tumours is thought to be participation in lipid synthesis [17]. In this study, an increase of ACE uptake, from non-significant with docetaxel to 40% with tamoxifen, to a decrease by 30% with doxorubicin, was observed. Hence, compared with other tracers, ACE seemed to have less utility for the monitoring of breast cancer treatment.

In the human body, choline is needed for the synthesis of phospholipids in cell membranes, methyl metabolism, transmembrane signalling, and lipid-cholesterol transport and metabolism. The uptake is transport mediated and intracellular choline is rapidly metabolised to phosphorylcholine (PC); it can also undergo acetylation to form acetylcholine or oxidation to form betaine (mainly in liver and kidney). The phosphorylation is catalysed by the enzyme choline kinase. After phosphorylation, the polar PC molecule is trapped within the cell. Various studies have revealed an increased choline uptake as well as an up-regulated activity of choline kinase and elevated levels of PC in cancer cells [18]. Although a primary use is in prostate cancer, it has also been suggested that CHO could be a potential PET tracer for imaging of breast cancer [19]. In this study, a 10 to 20% decrease of CHO uptake in MTS treated with doxorubicin and imatinib was observed. However, for doxorubicin treatment the decrease in uptake was much less than growth inhibition and for imatinib, the decrease was a false indication of treatment efficiency.

MET has been suggested as a tumour imaging agent [20-22]. The uptake of MET reflects increased amino acid transport and, in part, protein synthesis; it also is related to cellular proliferation activity. In MTS treated with paclitaxel, docetaxel and doxorubicin, MET uptake decreased slightly but to a lesser extent than the growth inhibition.

FLT has become one of the promising PET agents that reflect nucleoside transport into the cell and has been proposed as a marker for cellular proliferation [23-26]. FLT is phosphorylated by thymidine kinase-1 and then trapped intracellularly by entering the salvage pathway of DNA synthesis without incorporation into the DNA molecule. The FLT uptake was considerably decreased during 3 days of treatment with paclitaxel, docetaxel, doxorubicin and high dose of tamoxifen where also a growth inhibition was observed. The modest FLT uptake increase observed in imatinib treatment was correlated to the moderate growth enhancement. Ki67 staining illustrated and confirmed the anti-proliferative effect of the drugs monitored with FLT.

FDG is the most commonly used PET tracer in cancer diagnosis and has shown utility for treatment monitoring for selected agents. FDG uptake reflects glucose transporters expression and hexokinase in the cells, dual mechanisms that lead to fluorodeoxy-glucose-phosphate accumulating without further metabolism. It is the most commonly used PET tracer for diagnosis and treatment follow-up of breast cancer. FDG uptake reduced as a result of treatment with taxanes, doxorubicin [16] and the higher dose of tamoxifen. FDG uptake reduction correlated with growth inhibition, but not as clearly as FLT.

Our results showed high sensitivity of FLT, compared with FDG and other tracers, for monitoring the effects of the drugs in MTS. This is an example where PET tracers other than FDG seem necessary to improve the ability to measure and predict response and hopefully help to tailor therapy to individual patients. This study only used one cell line, MCF-7, and aimed primarily to show the methodology. To allow more general conclusions with respect to treatment monitoring of breast cancer, it would be necessary to explore further breast cancer cell lines, evaluate more concentrations of the drugs, investigate the time courses of drug effects by using pulse treatment, and probe more carefully the uptake pattern of the PET tracers.

There are also many additional factors that should be considered, e.g. concentrations of glucose, methionine and choline in the cell culture medium that are 5–10-fold higher compared to blood.

## Conclusion

This study shows the possibility of using MTS as a practical preclinical model to screen a range of PET tracers for breast cancer treatment monitoring. This, in combination with SASDM, improves the precision and allows accurate studies of the correlation between changes in PET tracer uptake and effects on growth of the MTS. We demonstrate the feasibility of *in vitro *quickly assessing a metabolic response profile of existing and newly developed anticancer drugs.

## Abbreviations

ACE = [1-^11^C]acetate; CHO = [^11^C]choline; FDG = 2-[^18^F]fluoro-2-deoxyglucose; FLT = [^18^F]3'-deoxy-3'-fluorthymifine; MET = [^11^C]methionine; MTS = multicellular tumour spheroid; PET = positron emission tomography; SASDM = semi-automated size determination method.

## Competing interests

The authors have applied for a patent on the automatic delineation of spheroid size method used in the study. The authors declare no other competing interests.

## Authors' contributions

AM and MB designed the study. AM performed the experiments and analysed the data and drafted the manuscript. MB, RJ and JC reviewed the results and manuscript and added important intellectual content to the manuscript. CB and BL reviewed and modified the manuscript. All authors participated in subsequent revisions of the manuscript and approved its final version.
